# Vagal predominance correlates with mood state changes of winter-over expeditioners during prolonged Antarctic residence

**DOI:** 10.1371/journal.pone.0298751

**Published:** 2024-07-05

**Authors:** Shiying Liu, Jianan Wang, Shaoling Chen, Jiamin Chai, Jigang Wen, Xuan Tian, Nan Chen, Chengli Xu

**Affiliations:** 1 Institute of Basic Medical Sciences, Chinese Academy of Medical Sciences, Beijing, China; 2 Pingxiang Third People’s Hospital, Pingxiang, Jiangxi, China; 3 Center of Environmental and Health Sciences, Chinese Academy of Medical Sciences, Beijing, China; Universidade Federal de Minas Gerais, BRAZIL

## Abstract

**Objective:**

Winter-over expeditioners in Antarctica are challenged by various environmental and psycho-social stress factors, which may induce psychophysiological changes. The autonomic nervous system (ANS) plays a crucial role in the adaptation process under stress. However, the relationship between ANS activity and the mood states of expeditioners remains largely unexplored. This study aims to uncover the pattern of ANS adjustment under extreme Antarctic environments and provide new insights into the correlations between ANS activity and mood state changes, which may provide scientific data for medical interventions.

**Methods:**

Fourteen expeditioners at Zhongshan Station participated in this study. The study was conducted during four representative periods: pre-Antarctica, Antarctica-1 (pre-winter), Antarctica-2 (winter), and Antarctica-3 (summer). The heart rate variability (HRV) of the expeditioners was continuously measured for 24 hours to evaluate ANS activity. Plasma levels of catecholamines were tested by ELISA. Mood states were assessed by the Profile of Mood States (POMS) scale.

**Results:**

HRV analysis showed a disturbance of ANS during winter and summer periods. For frequency domain parameters, very low frequency (VLF), low frequency (LF), high frequency (HF), and total power (TP) significantly increased during the second half of the mission. Especially, LF/HF ratio decreased during summer, indicating the predominance of vagal tone. Results of the time domain analysis showed increased heart rate variability during the austral winter and summer. Plasma epinephrine (E) significantly increased during residence in Antarctica. Compared with pre-Antarctica, the vigor, depression, and anger scores of the expeditioners decreased significantly during the austral summer. Notably, the depression score showed a moderate positive correlation with LF/HF, while weak negative correlations with other HRV indicators, including TP, VLF, and LF. Anger score showed a moderate positive correlation with LF/HF and weak negative correlations with the average normal-to-normal (NN) interval, and the root mean square of differences between adjacent RR intervals (RMSSD). Plasma E level weakly correlated with the average NN interval.

**Conclusion:**

Prolonged residence in Antarctica increased the ANS activities and shifted the cardiac autonomic modulation towards vagal predominance. The alteration of HRV correlated with mood states and plasma epinephrine levels.

## Introduction

Heart rate variability (HRV) is the variation between continuous cardiac cycles over time, and it reflects the heart’s adaptability to changing environments [1]. According to previous studies, HRV can not only be used to assess overall cardiac health, but also be regarded as the “gold standard” in the noninvasive assessment of the autonomic nervous system (ANS) homeostasis, reflecting the balance of sympathetic nervous system (SNS) and parasympathetic nervous system (PSNS) [2, 3].

Because there are no native residents in Antarctica, research is usually conducted among expeditioners to clarify the changing pattern of stress, adaptation, and injury process of humans under this extreme environment. However, research focused on the disturbance of ANS in Antarctica is still limited. Previous studies suggested that a relatively short-term sojourn of less than 60 days in Antarctica, during which expeditioners were accommodated in unheated summer huts or tents, did not significantly affect the homeostasis of the ANS [4, 5] or only triggered the activation of sympathetic tone during the first month upon arriving in Antarctica, followed by the gradual attenuation of SNS activity [6]. Interestingly, prolonged residence in Antarctica induced different patterns of heart rate variability compared to short-term sojourn. Winter-over expeditioners showed a balanced regulatory influence during the first half of the expedition, but an increase in sympathetic component at the end of the expedition [6–8]. However, all these studies are based on short-term resting HRV examination (5–10 min), which may not be able to represent processes with slow fluctuations (e.g., circadian rhythms) and the cardiovascular system’s response to a variety of environmental and social stress [9]. Although Farrace et al. evaluated the 24-hour electrocardiogram of Italian expeditioners and found a reduction of sympathetic outflow [10], the study only observed HRV alteration over 40 days during the austral summer. Therefore, the changing pattern of 24-hour HRV during prolonged residence in Antarctica remains largely unexplored.

Recent studies have uncovered the correlations between HRV and mood states [11]. For example, depressive mood was associated with the PSNS component of HRV [12, 13], while positive affect was negatively associated with LF power [14, 15]. According to previous reports, Antarctic winter-over expeditioners may experience mood fluctuations during the mission, some of them showed increased depression, stress, or anxiety [16–21], while others exhibited positive adaptation and emotions [22–24]. However, the relationship between mood state changes and alterations in autonomic function among winter-over expeditioners remains to be elucidated.

Chinese Zhongshan Station (69°22′24″ S, 76°22′40″ E) is located in the Antarctic Circle. Due to its geographical location, winter-over expeditioners have to cope with unique photoperiods, i.e., 58 polar nights during winter and 54 polar days during summer on average. During the 6-month wintering period from March to September, only a few winter-over expeditioners stay at Zhongshan Station in an isolated and restricted environment. Therefore, we speculate that expeditioners may experience dysregulation of cardiac autonomic homeostasis, accompanied by mood state alterations under the Antarctic extreme environmental and social stresses. In this study, we will evaluate the impact of Antarctic prolonged residence on the ANS and its key regulatory hormones, i.e., catecholamine, as well as explore potential relationships between HRV and mood state changes. This study may propose new insights into the pattern of ANS adjustment under the Antarctic extreme environment and provide clues for the interaction between HRV and mood states.

## Materials and methods

### Subjects

The study was approved by the ethics committee of Peking Union Medical College (ethic number: 023–2012) and conducted under the supervision of the Chinese Arctic and Antarctic Administration and the Polar Research Institute of China. All the participants gave their signed informed consent before the study.

**Fig 1:**
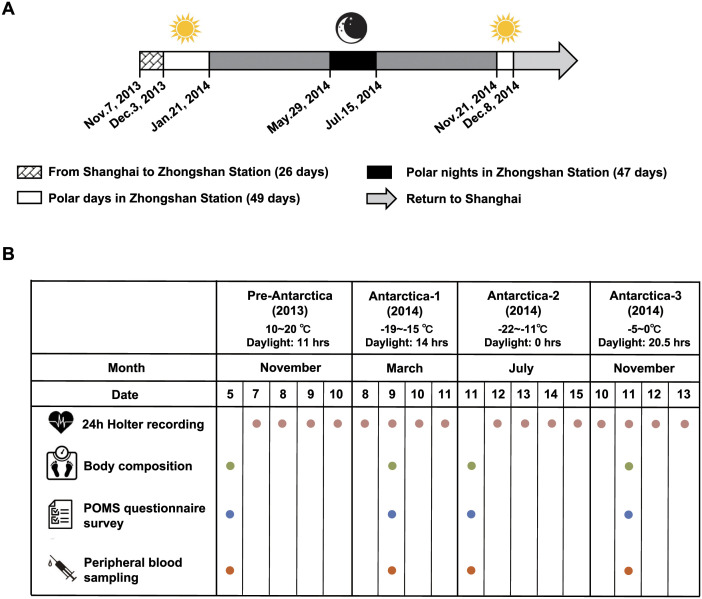
The itinerary and schedule of sampling and tests of the winter-over expeditioners of the Chinese Antarctic expedition during 2013–2014. A. The expedition team left Shanghai on Nov.7, 2013. After 26 days of voyage, they arrived at Zhongshan Station and stayed for 13 months before they returned to China. During the expedition, there were 49 polar days and 47 polar nights. B. All the examinations and blood sample collections were carried out on 4~5 consecutive days each time. The specific testing time is shown in the table. The circles represent the measurements made at each time point. n = 14.

The winter-over expedition team consisted of 18 men. Fourteen of them, aged 22–52 years old (average age was 29.63 ± 4.69 years old), participated in this study throughout the entire process. Their average height was 172.50 ± 8.06 cm (range from 160–185 cm). All individuals were in good mental and physical health and received physical examinations before the study. The crew consisted of the station leader, station administrator, doctor, scientists, and other workers. The expeditioners left from Shanghai (China) on November 7, 2013, by the Xuelong icebreaker ship, replenished supplies at Fremantle (Australia) on November 20, 2013, and arrived at Zhongshan Station on December 3, 2013. The summer team members left Zhongshan Station on February 26, 2014, leaving behind the winter-over expeditioners to reside for another ten months. In total, they spent thirteen months staying at Zhongshan Station and then left Antarctica in December 2014. Measures of body composition, mood state, HRV, and plasma catecholamine were carried out during four periods: pre-Antarctica, Antarctica-1 (pre-winter), Antarctica-2 (winter), and Antarctica-3 (summer). Because only five sets of devices were available, the 24-hour HRV recording was carried out on four consecutive days. Blood collecting, measures of body composition, and questionnaire surveys were accomplished on the same day. Details are shown in Fig 1.

### Environment of the station and lifestyle

Antarctic Zhongshan Station (69°22′24″ S, 76°22′40″ E) is located on Larsemann Hills in Prydz Bay and was established in 1989, with an average altitude of about 11 meters. The average temperature is -10 °C. The Zhongshan Station is equipped with automatic centrally heated permanent facilities and accommodates about 17–19 expeditioners during the austral winter. It is a self-contained unit with 15 buildings (office buildings, administrative buildings, scientific research buildings, and entertainment buildings) and the area is about 2700 m^2^. The indoor temperature is 16–20 °C. Outdoor activity during winter is limited to those essential for station maintenance and scientific activities. Adventure sports were strongly discouraged to reduce the risk of traumatic injuries. The lowest environmental temperature in 2014 was -29 °C. The polar days were 49 days (the sun did not set from December 3, 2013 to January 21, 2014) with an average temperature of -17 °C, and the polar nights were 47 days (the sun did not rise from May 29, 2014 to July 15, 2014) with an average temperature of 2 °C. The average daylight duration and daily temperature at Zhongshan Station during the expedition are shown in S1 Fig. The dataset is provided by the National Cryosphere Desert Data Center (http://www.ncdc.ac.cn, doi: 10.12072/ncdc.azs.db3011.2023).

### Examination of body composition

The body weight (kg), BMI (kg/m^2^), body fat (%), and body muscle (%) of expeditioners were measured by a body fat analyzer (HBF-701, OMRON, Japan) before and during the expedition, right after blood sample collection. This body fat analyzer can predict human body composition based on the bioelectrical impedance analysis (BIA). The principle of BIA is the differences in the electrical conductive properties of lean mass and fat mass [25]. Lean mass, consisting of water and electrolytes, is a good electrical conductor, while fat mass without water is a poor conductor [25].

### HRV recording and indicators

HRV examinations were conducted by the professional physician using Seer Light extend 12-channel Holter recorders (GE Marquette Mars800-Holter analyzer, Milwaukee, Wisconsin) with a sampling rate of 10000 Hz. The data collection usually started between 9:00~11:00 p.m., and the entire procedure lasted for 24 hours (1440 min). A 24-hour assessment was conducted since long-term measurements can reflect a more comprehensive interaction of autonomic outputs. Because only five sets of Holter recorders were available, the physician had to spend four consecutive days to complete the examination of fourteen volunteers. The participants were instructed to refrain from caffeine or tea consumption for at least three hours before the measurement and to avoid any strenuous exercise 24 hours prior to the examination.

During the data recording, participants were asked to behave normally without high-intensity activity. After the data collection procedure, HRV data were transferred to a computer via a USB link and analyzed using the Holter Analysis Workstation software. The software enabled visual examination for HRV analysis. The RR intervals were then examined, and the possible premature beats and artifacts (missed or spurious beats) were deleted or replaced with interpolations by an experienced physician. Recordings of more than 18 hours of usable data were included for analysis. To identify the spectral components, the software decomposed the signal into a series of sine waves of different amplitudes and sequences, using the Fast Fourier Transform mathematical device and trend elimination method.

Frequency domain parameters include ultra-low frequency (ULF, below 0.003 Hz, ms^2^), very low frequency (VLF, between 0.003 and 0.04 Hz, ms^2^), low frequency (LF, between 0.04 and 0.15 Hz, ms^2^), high frequency (HF, between 0.15 and 0.40 Hz, ms^2^), total power (TP, ms^2^), and the ratio of LF to HF (LF/HF). TP was calculated by combining the ULF, VLF, LF, and HF bands. The HF power, also known as the respiratory band, indexes parasympathetic activity and responds to respiratory oscillations [9]. LF is produced by SNS, PSNS, and baroreceptors, or by baroreflex activity alone in resting conditions [26–28]. However, since LF is the only parameter to assess SNS output, it is generally considered as an indicator describing SNS activity [2]. Therefore, LF/HF is often used to estimate the ratio between the SNS and PSNS activity. The origin of ULF is still controversial, some research suggested that slow-acting biological processes may contribute to these frequencies, including circadian rhythms, core temperature, metabolism, and the renin-angiotensin system [2, 26, 29]. VLF power may be generated by the intrinsic nervous system, physical activity, thermoregulatory, renin-angiotensin, and endothelial influences on the heart [30–33], and may reflect the parasympathetic outflow [31, 34].

Time domain parameters quantify the amount of HRV during the recording periods, which consist of average NN interval, SDNN, SDANN, SDNNI, and RMSSD. The average normal-normal (NN) interval is the average interval between adjacent normal sinus beats, resulting from sinus node depolarization. SDNN is the standard deviation of all the NN intervals of the HRV recording, and SDNN is highly associated with ULF, VLF, and LF power [35]. SDANN means the standard deviation of the averages of NN intervals for each 5-minute segment of the HRV recording. To obtain SDANN, we first calculated the average NN interval of every 5-minute segment during the recording and then calculated the standard deviation of the averaged values. SDNNI (SDNN index) is the average of the standard deviations of all NN intervals for each 5-minute segment of the 24-hour HRV recording. It is obtained by first calculating the standard deviation of every 5-minute segment during the recording, followed by calculating the average of these standard deviations. In the condition of a 24-hour measurement, SDNNI may be correlated to the VLF band [30]. RMSSD is the root mean square of differences between adjacent RR intervals and is the primary time-domain parameter used to index vagal outflow [30]. PNN50 is calculated as the percentage of adjacent NN intervals differing by more than 50 ms, and it is affected by PNS activity [35].

### Measurements of plasma catecholamine

The fasting venous blood samples, 5 mL for each participant, were collected at 7:00~8:00 a.m. using disposable vacuum blood tubes with anticoagulant EDTA, and then centrifuged (3000 rpm for 10 min at 4°C) to obtain plasma. The plasma was immediately transferred to 2 mL preservation tubes for storage at −80 °C. All blood samples collected at Zhongshan Station were transported via the Xuelong icebreaker ship in April 2015 at −40 °C to China for assay. The levels of plasma catecholamine, including epinephrine (E), norepinephrine (NE), and dopamine (DA), were detected by ELISA kits (RE59395, IBL International, Germany). According to the instructions of the manufacturer, 500 μL of each plasma sample was used for analyses and the measurements were performed in simplicate.

### Evaluation of mood states

Mood states of participants were assessed by the Chinese version of Profile of Mood States (POMS), a widely used self-report mood questionnaire consisting of 6 subscales (tension, depression, anger, vigor, fatigue, and confusion) with 65 items [36]. The questionnaires were collected between 9:00~10:00 a.m. on the same day of blood collection. The total mood disturbance (TMD) is calculated by adding the scores of negative moods (tension, depression, anger, fatigue, and confusion-bewilderment), and then subtracting the vigor score. The Chinese version of POMS has high test-retest reliability and internal consistency and has been used in evaluating Chinese Antarctica expeditioners’ psychological states [37, 38].

### Statistical analysis

All the data are expressed as mean ± SD and statistical analysis was conducted by IBM SPSS Statistics 21 software (IBM Inc., Chicago, USA). Data was tested on the normality by Shapiro–Wilk tests. Since we conducted tests for each participant at each time point repeatedly, data with normal distribution was analyzed using variance (ANOVA) for repeated measures followed by the Bonferroni method, including BMI, body fat, body muscle, VLF, HF, LF/HF, average NN interval, SDNN, SDANN, SDNNI, RMSSD, and vigor. Data with non-normal distribution was examined by using the Friedman rank-sum test for dependent variables, including body weight, ULF, LF, TP, PNN50, tension, depression, anger, fatigue, confusion, TMD, E, NE, and DA.

Repeated measures correlations between POMS scores, catecholamine, and HRV indicators were analyzed by Repeated measures correlation (rmcorr) to determine the common within-individual association for paired measures assessed on four occasions for multiple individuals using the “rmcorr” package of R software (V2021.09.1) to obtain correlation coefficients (r) and *P* values. The code used for repeated measures correlations analysis is provided in S1 File. The degree of correlation was determined according to the absolute magnitude of the observed correlation coefficient as follows: negligible, 0.00–0.10; weak, 0.10–0.39; moderate, 0.40–0.69; strong, 0.70–0.89; very strong, 0.90–1.00 [39]. *P*<0.05 was regarded as statistically significance.

## Results

### Anthropometric characteristics of participants

According to Table 1, body weight and body mass index (BMI) showed an obvious decrease during the austral summer [body weight: *χ*^*2*^ = 13.101, *P* = 0.004, *P* ^Antrctica-3^ = 0.024; BMI: *F* = 8.911, *P*<0.001, *P* ^Antrctica-3^ = 0.017]. However, both percent body fat and muscle showed no significant changes during the expedition (Table 1).

**Table 1:** Anthropometric characteristics of the expeditioners.

Parameters	Pre-Antarctica	Antarctica-1	Antarctica-2	Antarctica-3
Body weight (kg)	74.86 ± 12.82	74.19 ± 12.50	72.20 ± 13.41	70.26 ± 10.46*
BMI (kg/m^2^)	25.05 ± 2.95	24.76 ± 2.75	24.07 ± 2.87	23.48 ± 2.10*
Body fat (%)	16.80 ± 2.56	17.10 ± 2.66	16.81 ± 2.83	15.96 ± 2.62
Body muscle (%)	32.06 ± 1.67	31.81 ± 1.77	31.91 ± 1.90	32.42 ± 1.91

Body weight, BMI, body fat, and body muscle of expeditioners were measured in the morning at pre-Antarctica (November 2013), Antarctica-1 (March 2014, before austral winter), Antarctica-2 (July 2014, austral winter), and Antarctica-3 (November 2014, austral summer), following the blood sample collection.

*P<0.05 versus pre-Antarctica (mean±SD, n = 14).

BMI, body mass index.

### Changes in heart rate variability of winter-over expeditioners

Compared with pre-Antarctica, TP, VLF, LF, and HF increased during austral winter and summer, while ULF was unaltered (Table 2) [TP: *χ*^*2*^ = 11.743, *P* = 0.008, *P* ^Antrctica-2^ = 0.032, *P* ^Antrctica-3^ = 0.004; VLF: *F* = 8.119, *P*<0.001, *P* ^Antrctica-2^ = 0.018, *P* ^Antrctica-3^ = 0.032; LF: *χ*^*2*^ = 12.343, *P* = 0.006, *P* ^Antrctica-2^ = 0.040, *P* ^Antrctica-3^ = 0.002; HF: *F* = 6.411, *P* = 0.001, *P* ^Antrctica-2^ = 0.013, *P* ^Antrctica-3^ = 0.006]. On the contrary, LF/HF was significantly lower in austral summer than pre-Antarctica, which indicates the increase of HF was greater than LF (Table 2) (*F* = 3.785, *P* = 0.046, *P* ^Antrctica-3^ = 0.022). As for time domain parameters, average NN interval, SDNNI, and RMSSD were significantly higher during the second half of the expedition (Table 2) [average NN interval: *F* = 14.786, *P*<0.001, *P* ^Antrctica-2^ = 0.007, *P* ^Antrctica-3^ < 0.001; SDNNI: *F* = 7.470, *P*<0.001, *P* ^Antrctica-2^ = 0.013, *P* ^Antrctica-3^ = 0.010; RMSSD: *F* = 4.932, *P* = 0.005, *P* ^Antrctica-2^ = 0.031, *P* ^Antrctica-3^ = 0.033]. SDNN, SDANN, and PNN50 showed no significant changes (Table 2).

**Table 2:** HRV parameters at different time points.

	Pre-Antarctica	Antarctica-1	Antarctica-2	Antarctica-3
Frequency domain indicators				
TP (ms^2^)	46.26 ± 13.40	48.73 ± 13.25	53.82 ± 13.13*	53.07 ± 12.84**
ULF (ms^2^)	18.57 ± 5.14	19.52 ± 4.51	20.54 ± 4.73	20.72 ± 4.94
VLF (ms^2^)	33.06 ± 10.12	34.89 ± 9.61	38.77 ± 9.18*	37.75 ± 8.70*
LF (ms^2^)	22.85 ± 6.60	23.98 ± 7.24	26.67 ± 6.90*	26.32 ± 6.96**
HF (ms^2^)	12.55 ± 5.55	13.59 ± 5.28	15.52 ± 6.19*	15.72 ± 6.35**
LF/HF	1.99 ± 0.57	1.84 ± 0.39	1.82 ± 0.42	1.79 ± 0.46*
Time domain indicators				
Average NN interval (ms)	757.29 ± 71.16	786.86 ± 75.32	834.50 ± 84.48**	836.57 ± 63.01**
SDNN (ms)	142.00 ± 33.76	152.29 ± 35.59	150.93 ± 35.48	160.86 ± 35.69
SDANN (ms)	131.29 ± 34.21	136.86 ± 31.07	131.07 ± 34.48	144.57 ± 36.26
SDNNI (ms)	58.21 ± 16.51	61.79 ± 16.81	67.86 ± 16.69*	66.86 ± 15.92*
RMSSD (ms)	29.36 ± 10.79	31.57 ± 12.12	35.86 ± 13.21*	35.29 ± 13.59*
PNN50 (%)	8.84 ± 7.44	10.04 ± 7.96	13.07 ± 8.49	12.49 ± 9.33

24-hour HRV recordings were conducted by professional physicians at pre-Antarctica (November 2013), Antarctica-1 (March 2014, before austral winter), Antarctica-2 (July 2014, austral winter), and Antarctica-3 (November 2014, austral summer).

*P<0.05 versus pre-Antarctica,

**P<0.01 versus pre-Antarctica (mean±SD, n = 14).

TP, total power; ULF, ultra-low frequency; VLF, very low frequency; LF, low frequency; HF, high frequency; LF/ HF, the ratio between LF and HF; average NN interval, average normal-to-normal interval; SDNN, standard deviation of all the NN intervals of the HRV recording; SDANN, standard deviation of the averages of NN intervals for each 5 min segments of the HRV recording; SDNNI, average of the standard deviations of all NN intervals for each 5 min segments of the HRV recording; RMSSD, root mean square of differences between adjacent RR intervals; PNN50, percentage of adjacent NN intervals differing by more than 50 ms.

### Plasma epinephrine levels of expeditioners increased during the expedition

**Fig 2:**
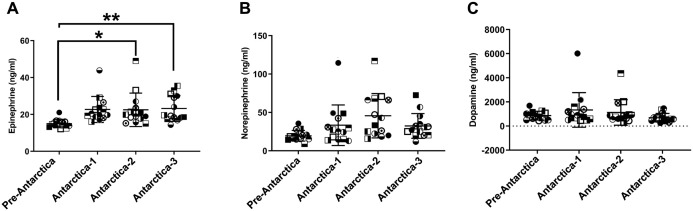
The level of plasma catecholamine of winter-over expeditioners at different time points. Plasma epinephrine (A), norepinephrine (B), and dopamine (C) levels were detected according to the instructions of ELISA kits. Increments in epinephrine levels were observed at Antarctica-2 and Antarctica-3, while norepinephrine and dopamine levels showed no significant changes. Pre-Antarctica, November 2013; Antarctica-1, March 2014, before austral winter; Antarctica-2, July 2014, austral winter; Antarctica-3, November 2014, austral summer. *P<0.05 versus pre-Antarctica, **P<0.01 versus pre-Antarctica (mean±SD, n = 14).

Plasma E levels increased significantly during the prolonged residence in Antarctica compared to pre-Antarctica (Fig 2A) (*χ*^*2*^ = 18.771, *P*<0.001, *P* ^Antrctica-2^ = 0.038, *P* ^Antrctica-2^ = 0.004). NE and DA levels were unaltered during the expedition (Fig 2B and 2C).

### Changes in mood states of winter-over expeditioners

**Fig 3:**
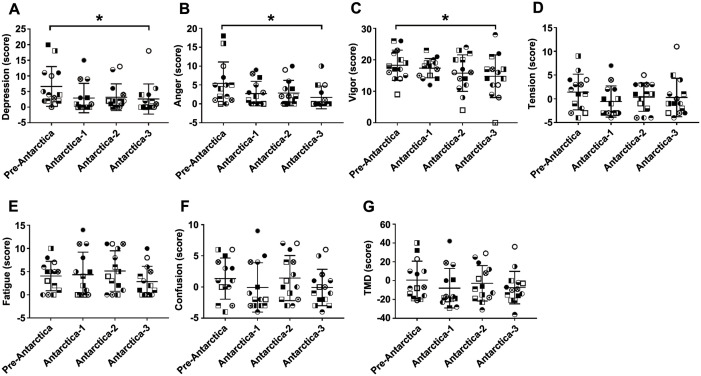
Changes in POMS scores of the winter-over expeditioners. Scores of the seven subscales of POMS were calculated according to the official scoring methods. Depression (A), anger (B), and vigor (C) scores increased during the austral summer, while other subscale scores were unaltered (D-G). *P<0.05 versus pre-Antarctica (n = 14). POMS, Profile of Mood States; TMD, total mood disturbance.

According to Fig 3, compared with pre-Antarctica, scores of vigor, depression, and anger decreased significantly during the austral summer [vigor: *F* = 3.673, *P* = 0.020, *P* ^Antrctica-3^ = 0.035; depression: *χ*^*2*^ = 7.34, *P* = 0.047, *P* ^Antrctica-3^ = 0.036; anger: *χ*^*2*^ = 8.632, *P* = 0.035, *P* ^Antrctica-3^ = 0.032]. Other subscales of POMS (tension, fatigue, confusion, and TMD scores) showed no significant changes (Fig 3).

### HRV parameters are correlated with mood states and plasma epinephrine level

**Fig 4:**
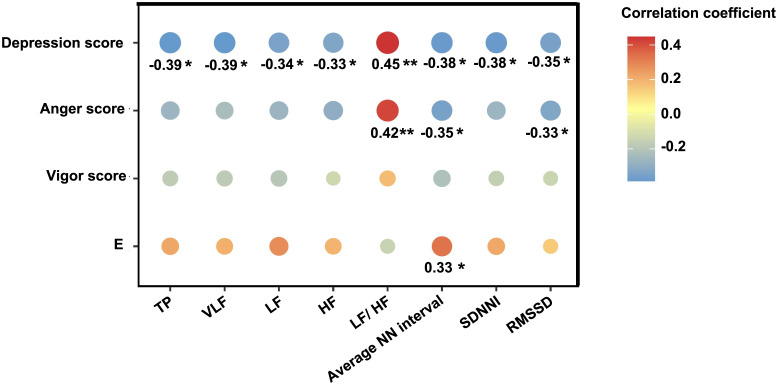
HRV parameters are correlated with mood states and plasma epinephrine level. Repeated measures correlations were analyzed to determine the common within-individual association for paired measures assessed on four periods for multiple individuals. Only correlation coefficients with significance were presented. *P<0.05, **P<0.01. TP, total power; VLF, very low frequency; LF, low frequency; HF, high frequency; LF/ HF, the ratio between LF and HF; average NN interval, average normal-to-normal interval; SDNNI, average of the standard deviations of all NN intervals for each 5 min segments of the HRV recording; RMSSD, root mean square of differences between adjacent RR intervals; E, epinephrine.

According to Fig 4, TP, VLF, LF, HF, average NN interval, SDNNI, and RMSSD were negatively associated with depression scores with weak correlation coefficients, while there was a moderate positive correlation between LF/HF and depression scores. Anger score was negatively and weakly correlated with average NN interval and RMSSD, while moderately correlated with LF/HF (Fig 4). We also found a weak positive correlation between E and average NN interval (Fig 4).

## Discussions

ANS plays a leading role in the adaptation of humans to a variety of stresses, and the assessment of the activity of both the SNS and PSNS branches is very important for the characterization of the adaptive processes [1, 2]. So far, HRV monitoring is considered one of the most reliable, informative, and applicable approaches to evaluating ANS activity [2, 26]. In this study, we found that healthy expeditioners who resided in Antarctica for more than a year experienced significant increases in sympathetic and vagal outflow, with the predominance of vagal modulation at the end of the mission. Interestingly, the depression degree of expeditioners significantly correlated with all the changed HRV indicators. These results depict the changing pattern of cardiac autonomic regulation during prolonged residence in Antarctica and may further provide novel clues for the interplay between HRV and mood states.

A recent study suggested that short-term HRV measurement is not interchangeable with 24-hour recording, as their physiological meaning may be profoundly different [9]. Despite this, we observed balanced cardiac sympathovagal regulation during the early stages of the expedition and increased LF and plasma epinephrine levels upon the end of the mission, which were consistent with previous reports based on 10-minute HRV recordings [6–8]. To our surprise, parameters reflecting parasympathetic activity also significantly increased after about half of the mission. Especially, we observed the reduction of LF/HF during the austral summer period, indicating the predominance of vagal modulation. These results seem to contradict previous findings [6–8]. However, this is understandable considering the differences in experimental design between researches. Previous studies collected resting HRV for 10 minutes with subjects in a supine position in the research station at a suitable temperature [6–8]. By contrast, in this study, we examined the HRV of subjects continuously for 24 hours, during which subjects were asked to avoid high-intensity activity but carried out their work as normal, including fieldwork with protective clothing. The environmental stress factors during overwintering in Antarctica, for example, changed photoperiod and confinement, are similar to space travel. Intriguingly, our results are in line with observations reported in a 105-day simulated mission to Mars. Vigo et al. obtained 24-hour electrocardiogram records of crew members during the mission, and discovered increased amplitude of VLF, LF, and HF during wake periods, while LF/HF decreased, suggesting augmented parasympathetic predominance [40]. Therefore, the changing pattern of autonomic nervous activity reported in our study may also contribute to the psychophysiological adaptation under other environments with similar stimuli. Cardiac autonomic modulation can be affected by multiple factors, such as the respiratory system, endocrine, circadian rhythm, health, and mood state [9]. Therefore, the alteration of HRV in Antarctica can be interpreted as the overall effect under complicated environmental-psychological stimulations.

One of the factors that may affect cardiac autonomic homeostasis is mood state. Especially, we found weak to moderate correlations between depression and HRV. Recent studies uncovered the role of HRV in reflecting the efficiency of the prefrontal cortex in modulating emotional reactivity, psychological flexibility, and social engagement [41]. According to previous reports, individuals with depression had lowered HRV and reduced vagal modulation, showing a decrease in RMSSD, LF, and HF and an increase in LF/HF [42–49]. Kemp et al. and Lee et al. suggested that the severity of depression and subsyndromal depressive symptoms are negatively associated with HRV [44, 49]. In our study, we observed a decrease in depression during the end of the expedition, which can be interpreted by the cancellation of restrictions on free activities and the relieving of psychological stresses after long-term wintering. Along with the improved mood states, the autonomic nervous regulation of expeditioners shifted towards vagal dominance. In accordant with previous reports, we also found depression score showed weak negative correlations with most HRV indicators, except for a moderate positive correlation with LF/HF. In summary, the changes in HRV, at least to some extent, may reflect the fluctuations in the mood states of winter-over expeditioners, especially the degree of depression.

Another reason that may contribute to the autonomic changes is the disturbance of sleep and circadian rhythm. Many studies have reported that winter-over expeditioners experienced exacerbated sleep fragmentation, decreased slow wave sleep (SWS), and delayed melatonin secretion during austral winter and summer, primarily due to the changed photoperiods [19, 50–57]. Previous studies suggested that shift work is associated with modifications of the cardiac autonomic profile. Despite variations in study subjects and designs, most of the research found that shift workers showed depressed vagal activity and increased cardiac sympathetic tone, according to the lower HF and RMSSD accompanied by higher LF and LF/HF [58–63]. On the contrary, implementing an ergonomic work schedule among shift workers had positive impacts on the maintenance of ANS homeostasis [64]. Furthermore, an increase in sympathetic activity was observed in insomniacs [63, 65–70], which could be reversed after gabapentin or cognitive-behavioral treatment [69, 70]. Therefore, during the austral winter and summer, the increase in sympathetic activity of expeditioners could be partially interpreted by disrupted circadian rhythm and sleep. Optimizing the daily schedules of expeditioners or using blue light exposure to improve sleep may help sustain the homeostasis of ANS during overwintering.

Studies suggested that regular physical activity had a positive effect on autonomic control of the heart in adults by increasing the HRV [71]. Overweight and obese subjects have higher sympathetic and lower parasympathetic activity, and sedentary time is negatively correlated with HF while positively correlated with LF and LF/HF [72]. Conversely, exercise training can help improve cardiac autonomic nervous function, significantly increasing SDNN, TP, LF, and HF while reducing LF/HF [73–75]. Therefore, regular physical activity may activate both sympathetic and parasympathetic branches but has a stronger effect on the vagal nervous system. Our study also observed a reduction in the body weight of expeditioners during the austral summer, which could be due to the increased workload before they left the station, such as cleaning up the snow in the station area, vehicle ex-warehousing, organizing the observation data, and so on. However, given that the percent body fat and muscle showed no significant changes, we suppose the intensity of physical activity may contribute to the PSNS dominance during the austral summer with limited effects.

Although our study is scarce and valuable, there are still some limitations. The sample size is relatively small, whereas it is comparable to other research conducted among Antarctic expeditioners. Yet, our study period spanned over one year, and all the volunteers lived in the same environment with similar lifestyles and diets, which eliminated the influence of some confounding factors.

In conclusion, this study revealed dynamic change patterns of mood states and activity of the autonomic nervous system of winter-over expeditioners in Antarctica. The correlation between negative moods and HRV found in our study may provide scientific data for deciphering the interplay between psychological states and ANS outputs.

## Supporting information

S1 Fig The daylight duration and environmental temperature at Zhongshan Station during the expedition. A. The daylight durations were calculated based on the sunrise and sunset time of each day during the expedition. B. The change curve of the average environmental temperature of Zhongshan Station was illustrated according to the conventional meteorological observation dataset provided by the National Cryosphere Desert Data Center.
(EPS)

S1 File The R code of repeated measures correlations analysis. Repeated measures correlations between POMS scores, catecholamine, and HRV indicators were analyzed by Repeated measures correlation (rmcorr) to determine the common within-individual association for paired measures assessed on four occasions for multiple individuals using the “rmcorr” package of R software (V2021.09.1) to obtain correlation coefficients (r) and *P* values.
(DOCX)

## Abbreviations

ANS: Autonomic nervous system
Average NN interval: Average normal-to-normal interval
BIA: Bioelectrical impedance analysis
BMI: Body mass index
DA: Dopamine
E: Epinephrine
HF: High frequency
HRV: Heart rate variability
LF: Low frequency
NE: Norepinephrine
PNN50: Percentage of adjacent NN intervals differing by more than 50 ms
POMS: Profile of Mood States
PSNS: Parasympathetic nervous system
RMSSD: Root mean square of differences between adjacent RR intervals
SDANN: Standard deviation of the averages of NN intervals for each 5 min segments of the HRV recording
SDNN: Standard deviation of all the NN intervals of the HRV recording
SDNNI: Average of the standard deviations of all NN intervals for each 5 min segments of the HRV recording
SNS: Sympathetic nervous system
TMD: Total mood disturbance
TP: Total power
ULF: Ultra-low frequency
VLF: Very low frequency
