# Supplementary material for: Vagal predominance correlates with mood state changes of winter-over expeditioners during prolonged Antarctic residence
Source: PLoS One. 2024 Jul 5;19(7):e0298751. doi: 10.1371/journal.pone.0298751 (PMC11226091; doi:10.1371/journal.pone.0298751)
Supplement: S1 File — Repeated measures correlations between POMS scores, catecholamine, and HRV indicators were analyzed by Repeated measures correlation (rmcorr) to determine the common within-individual association for paired measures assessed on four occasions for multiple individuals using the “rmcorr” package of R software (V2021.09.1) to obtain correlation coefficients (r) and P values. (DOCX) [file pone.0298751.s002.docx]

**Supplementary material 1. The R code for repeated measures correlations analysis**

install.packages("rmcorr")

library(rmcorr)

data <- read.csv("correlation analysis.csv")

Y<-colnames(data[3:10]) # columns of changed HRV parameters, including TP, VLF, LF, HF, LF/ HF, average NN interval, SDNNI, and RMSSD

X<-colnames(data[11:14]) # columns of other changed parameters, including depression score, anger score, vigor score, and epinephrine

cor <- function(participant = "ID",

measure1 = "a",

measure2 = "b",

dataset = data){

mycor <- rmcorr(participant,

measure1, measure2, dataset)

col1 = c(col1,measure1); col2 = c(col2, measure2); col3 = c(col3,mycor$r);col4 = c(col4,mycor$df);col5 = c(col5,mycor$p)

filted = data.frame(col1,col2,col3,col4,col5)

return(filted)

}

filted = data.frame(col1 = NA, col2 = NA, col3 = NA,col4 = NA,col5 = NA)

col1 = NULL ;col2 = NULL;col3 = NULL;col4 = NULL;col5 = NULL

data$ID=as.factor(data$ID)

for (i in 1:length(Y)){

for (j in 1:length(X)){

one <- cor(participant = "ID", measure1 =Y[i], measure2 =X[j], dataset = data)

filted = rbind(filted, one)

}

}

filted = filted[-1,]

colnames(filted) = c("a","b","r","df", "p")

# filted = filted[filted$p <= 0.05,]

filted = unique(filted)

library(reshape2)

r <- dcast(filted, a~b,value.var = "r")

p <- dcast(filted, a~b,value.var = "p")

write.table(r,file="r-HRV-other parameters.txt",sep="\t",na="",row.names = T,col.names = T)

write.table(p,file="p-HRV-other parameters.txt",sep="\t",na="",row.names = T,col.names = T)
